# The impact of parent-child relationship on adolescent social adjustment following childhood abuse: the moderation of the serotonergic multilocus genetic variation

**DOI:** 10.3389/fpsyt.2026.1887601

**Published:** 2026-08-04

**Authors:** De Tang, Jiayi Ding, Hao Rao, Hanyun Cao

**Affiliations:** 1 School of Educational Science, Hunan Normal University, Changsha, China; 2 Art Education Center, Hunan University, Changsha, China; 3 Research Centre for Mental Health Education of Hunan Province, Changsha, China; 4 Cognition and Human Behavior Key Laboratory of Hunan Province, Changsha, China; 5 Department of Human Resources, Hunan Normal University, Changsha, China

**Keywords:** aggressive behavior, creativity, depressive symptoms, differential susceptibility model, stress sensitivity model

## Abstract

**Introduction:**

Childhood adverse experiences appear to increase sensitivity to stress, potentially influencing lifelong social adjustment. Previous studies suggest that serotonergic multilocus genetic variation moderates the relationship between environmental factors and social adjustment.

**Methods:**

This study investigated an adolescent sample (mean age 14.15 ± 0.63 years; N = 700), using theory-driven multilocus genetic profile scores (MGPS) to measure serotonergic multilocus genetic variation. Leveraging longitudinal data, we examined the interactions between serotonergic MGPS, childhood abuse (CA), and parent-child relationship and their impact on three adolescent adjustment outcomes: depressive symptoms, aggressive behavior, and creativity.

**Results:**

The findings revealed significant gene × environment × environment (G × E × E) interactions among serotonergic MGPS, CA, and parent-child relationship that predicted depressive symptoms in the mother-child model and aggressive behavior in both father-child and mother-child models. In contrast, creativity was predicted by a significant gene × environment (G × E) interaction between serotonergic MGPS and parent-child relationship, whereas no significant G × E × E interaction was found.

**Conclusions:**

This study highlights that adolescent social adjustment may reflect the interplay of genetic factors (serotonergic MGPS), distal experiences (CA), and proximal environmental influences (parent-child relationship), providing new insights into the mechanisms underlying adolescent social adjustment through the lens of gene-environment interactions.

## 1 Introduction

Social adjustment broadly refers to adolescents’ emotional, behavioral, and cognitive adaptation within social environments to attain developmentally appropriate goals (1). In the present study, adolescent social adjustment were operationalized as depressive symptoms, aggressive behavior, and creativity. Specifically, depressive symptoms (internalizing adjustment) and aggressive behavior (externalizing adjustment) are indicative of social maladjustment (2, 3). In contrast, creativity was included as an exploratory positive cognitive-adaptive outcome related to adolescent adjustment. Although creativity is conceptually distinct from social adjustment, it reflects cognitive flexibility, originality, and problem-solving capacity, which may contribute to positive development (4, 5).

Research consistently shows that positive and healthy interpersonal relationship enhances adolescents’ social adjustment and well-being (6, 7). Negative relationship, especially dangerous or hostile ones, can lead to negative cognitive schemas and self-evaluations, increasing the risk of psychological crises (8, 9). While multiple factors influence the risk of social adjustment disorders, the interaction between genetics and the environment, which has garnered attention from researchers in psychology, psychiatry, and epigenetics (10–12). This study aims to explore the moderating role of serotonergic multilocus genetic profile scores (MGPS) in the relationship between various forms of environmental stress (proximal and distal) and adolescent social adjustment. We assess serotonergic multilocus genetic variation and measure key environmental risks, such as the parent-child relationship and childhood abuse (CA), to examine their effects on depressive symptoms, creativity, and aggressive behavior.

### 1.1 Primary interpersonal relationship: parent-child relationship

Vrshek-Schallhorn et al. (13) emphasized the crucial importance of selecting appropriate candidate environments when investigating G × E effects on adolescent social adjustment. Studies indicate that adolescents with high genetic risk exhibit a stronger association between major interpersonal stressors, rather than minor or non-interpersonal stressors, and social adjustment (14). Bronfenbrenner’s ecological systems theory (2000) (15) highlights the role of external environments in individual development. The theory proposes that development results from the interaction between individuals and their surrounding environments. Parent-child relationship, as a core element of the family ecology, has long been a focus of research in developmental psychology, counseling, and practice (16).

In Chinese culture, which values interdependence, kinship, and interpersonal harmony (17), parent-child relationship has an even greater impact on adolescent social adjustment (18). Furthermore, a meta-analysis by Van Dijk et al. (19) on the relationship between parent-child interactions and adolescent social adjustment found that high-quality relationship, characterized by intimacy, secure attachment, and positive communication, enhance children’s self-esteem and life satisfaction. On the other hand, those from problematic family environments frequently struggle with depression, aggressive behavior, and various other psychological problems (20).

### 1.2 Interaction between environments: the stress sensitization model

The stress sensitization model, which posits that early stress exposure heightens sensitivity to future stressors, thereby impacting adolescent social adjustment (21), can be described as an interaction between environments (environment × environment, E × E). Research indicates that childhood adversities, such as child abuse, parental loss, and parental separation, significantly increase sensitivity to subsequent proximal stressors, elevating the risk of social adjustment like depression (22), insomnia (23), self-harm (24), and aggressive behavior (25). A large-scale study examining childhood adversity and mental disorders (n = 34,653) revealed that American adults with a history of childhood adversity had a stronger correlation between proximal stress events and depression, anxiety, and PTSD compared to those without such a history (26). Additionally, adolescents who experienced CA were found to have a higher risk of depression when facing recent low-intensity stress (27). Research also shows that people who experienced CA and neglect exhibit a reduced capacity to care for others, becoming less altruistic, which negatively affects their creativity (28).

### 1.3 Genetic variations in stress sensitivity

Numerous studies have confirmed the significant influence of genetic factors on adolescent social adjustment (29, 30). Genetic variations can markedly increase the risk of depression following exposure to childhood adversity or proximal stressors (31) and also affect creativity (32, 33). However, the simple gene-environment (G × E) model does not fully capture the complex etiology of adolescent social adjustment due to the interactive effects of stress across different developmental stages. Researchers have proposed a more intricate model where genetic variations moderate stress sensitivity resulting from childhood adversity. This model, suggesting that differences in social adjustment result from the interaction of genes, childhood adversity, and proximal stressors, is termed gene × environment × environment (G × E × E), with the first “E” referring to childhood adversity and the second to proximal stressors (34).

In a study exploring the G × E × E effects on depressive symptoms, Grabe et al. (35) found that the interaction among the serotonin transporter gene, CA, and abuse significantly predicted depressive symptoms in a sample of 1,974 Caucasian participants from Germany. Prior studies have proposed several possible biological pathways that may link serotonergic variation, childhood adversity, and stress sensitivity. For instance, childhood adversity has been proposed to influence serotonin-related biological processes, including receptor sensitivity and transporter availability, which may be relevant to mood regulation and depression risk (36, 37).

### 1.4 Beyond single candidate genes: a multi-locus approach

The differential susceptibility model (38) suggests that “plasticity” genes can produce effects akin to the saying “ One who mixes with vermilion will turn red; one who touches ink will be stained black.” People with these genes may face increased risks in adverse environments but might thrive in favorable ones. This perspective is supported by existing research (39, 40), indicating that genetic variation moderates the impact of early adversity on proximal stress sensitivity through G × E × E interaction effects, mainly focusing on the serotonin and HPA axis system.

In a longitudinal study investigating the corticotrophin hormone receptor (CRHR1) gene, which is linked to the HPA axis, findings showed that the A allele is associated with greater stress sensitivity following childhood adversity (41). Compared to the controversies surrounding single-gene studies, MGPS, which aggregate unweighted putative sensitivity alleles from *a priori* selected genetic variants (14), offer greater testing power and robustness. MGPS, which consider genetic information from multiple loci, are more suitable for studying complex traits involving numerous gene-environment interactions (34).

### 1.5 Serotonergic multilocus genetic variation

The serotonin system is one of the primary physiological systems involved in stress response. Dysregulation and dysfunction of the serotonin system can significantly impact an individual’s emotions, memory, and sleep, making it a key candidate for studies on depression, self-harm, and prosocial behavior (34, 42). Research indicates that childhood adversity can affect physiological changes in the serotonin system, such as cortisol levels, stress reactivity, and circadian rhythms (43, 44). These changes can increase the risk of depression following recent stress exposure (45).

In a study investigating the serotonergic MGPS and its interaction with interpersonal stress on depression, Vrshek-Schallhorn et al. (13) discovered that serotonergic MGPS significantly predicted adolescent depression when combined with interpersonal stress in two independent samples. This result was also confirmed in a third sample (14). Moreover, a recent systematic review of seven studies (including 3,585 participants) on genetic markers of stress occurrence demonstrated that the HPA axis, serotonin, and oxytocin system polygenic cumulative genetic risks can predict individual psychosocial susceptibility and moderate the effects of proximal life events (46). Additionally, Sotiropoulos and Anagnostouli (47) suggested a genetic foundation for serotonin system genes in artistic creativity, encompassing dance, music, and visual arts.

Based on the stress sensitization model and the differential susceptibility model, the present study examined G × E × E interaction effects of CA, parent-child relationship, and serotonergic MGPS on adolescent social adjustment. Specifically, the interaction between CA and parent-child relationship reflects an environment × environment (E × E) stress sensitization process, whereby early adverse experiences heighten adolescents’ sensitivity to subsequent proximal stressors (21). Furthermore, a MGPS approach was used to measure serotonergic multilocus genetic variation that may moderate the susceptibility to environmental influences on social adjustment, consistent with the differential susceptibility model (38). The present study deepens understanding of how distal environmental adversity, proximal relational experiences, and genetic sensitivity jointly shape adolescent social adjustment.

### 1.6 Current research

**Figure 1:**
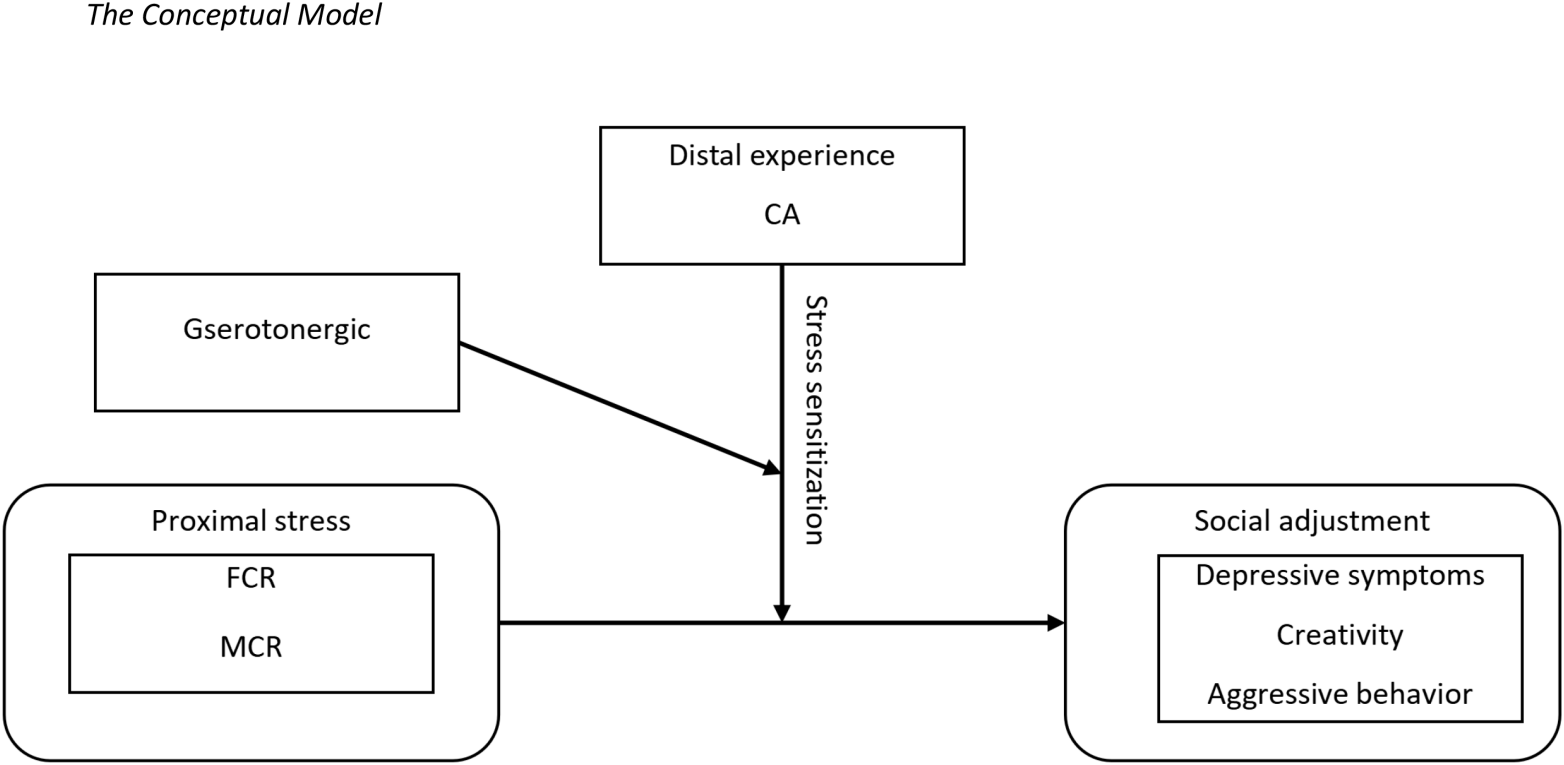
The conceptual model.

This study examined how genetic and environmental factors jointly relate to adolescent social adjustment outcomes. Specifically, we assessed three adolescent adjustment outcomes: depressive symptoms, aggressive behavior, and creativity. Given the limited literature linking serotonergic variation to creativity, analyses involving creativity were treated as exploratory. Using longitudinal data, we examine the role of serotonergic MGPS as a moderating factor between stress sensitization and adolescent social adjustment (See **Figure 1**). Based on the stress sensitization model, we examined whether retrospectively reported CA moderated the association between parent-child relationship and adolescent social adjustment, and whether this pattern further varied by serotonergic MGPS. Consistent with the differential susceptibility model, we expect that adolescents with higher serotonergic MGPS scores and high levels of parent-child relationship will exhibit lower depressive symptoms and aggressive behavior, as well as higher creativity, and vice versa.

## 2 Methods

### 2.1 Participants and procedure

The study participants were adolescents from Hunan Province, China. The first wave of measurements was conducted during the third week of November 2021. This phase assessed proximal stress factors (parent-child relationship) and collected genetic samples (buccal epithelial cells). The follow-up took place in the third week of October 2022, measuring distal environment (CA) and adolescent social adjustment (depressive symptoms, creativity, and aggressive behavior). CA was measured retrospectively at the second wave, as it is conceptualized as a relatively stable distal adverse experience occurring before adolescence (26). While retrospective reporting may entail recall bias, this approach is widely adopted in developmental psychopathology research on childhood adversity (48). However, because CA was not assessed prospectively before Wave 1, the temporal ordering among CA, parent-child relationship, and adolescent social adjustment should be interpreted cautiously. In light of these considerations, we refrain from drawing definitive causal interpretations.

At baseline (Wave 1, 2021), 863 adolescents completed questionnaires and provided genetic samples. At Wave 2 (2022), valid matched data were obtained for 700 participants, resulting in an overall attrition rate of 18.89%. The main reasons for attrition included school transfer, absence on survey days, and refusal to participate in the follow-up assessment. Independent-samples t tests and chi-square tests showed no significant baseline differences between retained and attrited participants in gender, only-child status, family SES, parental marital status, father-child relationship (FCR), mother-child relationship (MCR), or serotonergic MGPS (ps >.05). The final matched sample included 700 participants (mean age at the first measurement: 14.15 ± 0.63 years). This group included 320 females (45.7%) and 380 males (54.3%); 323 (46.2%) were only children, and 377 (53.8%) had siblings. Parental marital status included 583 (83.3%) from first marriages, 28 (4.0%) from divorced parents, 48 (6.8%) from remarried parents, and 41 (5.9%) from other types. Family economic status was reported as very good by 125 (17.9%), good by 414 (59.0%), average by 148 (20.2%), poor by 8 (1.2%), and very poor by 5 (0.7%).

In previous G × E × E studies, the interaction effect (R^2^) of serotonergic MGPS and environmental factors on social adjustment ranged from 0.02 to 0.04 (14, 42). With 700 participants and α = 0.05, the main regression analysis testing the interaction effects has an estimated power range of 96% to 100%. The study received approval from the institutional ethics committee. All experimental procedures obtained informed consent from schools, students, and their parents. Participants were compensated with notebooks and pens for their participation.

### 2.2 Instrument

#### 2.2.1 Parent-child relationship

We employed the Parent-Child Intimacy Questionnaire developed by Buchanan et al. (49) to assess the relationship between father and child, as well as mother and child. Each section consisted of 9 items (e.g., “Do you feel comfortable and natural when expressing your emotions to your father/mother?”). The questionnaire utilized a 5-point scale, ranging from 1 (“completely disagree”) to 5 (“completely agree”). Higher scores indicated a closer relationship between the adolescent and their father or mother. This instrument is applicable to Chinese adolescents (50). In our study, the Cronbach’s α coefficient for the FCR section was 0.92, and for the MCR section, it was also 0.92.

#### 2.2.2 CA

We used the Childhood Abuse Scale developed by Aslund et al. (51) to assess adolescent CA. With formal approval from Aslund, the scale was translated into Chinese using the back-translation method by two doctoral students specializing in English. This translation was then evaluated by a panel of ten experts, including psychology professors, doctoral candidates, and psychologists. Two items that were not contextually appropriate for the Chinese setting were removed, resulting in a final scale of seven items (e.g., “Did your parents ever push, hit, or use any other form of violence against you?”). Responses were scored on a scale from 0 (“never or rarely”) to 1 (“at least once a year”), with total scores ranging from 0 to 7. In this study, the Cronbach’s α coefficient for the childhood abuse scale was 0.72.

#### 2.2.3 Depressive symptoms

We employed the Short Form of the Center for Epidemiological Studies Depression Scale (SF-CES-D), developed by Andresen et al. (52), to assess depressive symptoms. This scale has shown strong applicability among Chinese adolescents (53). It consists of 10 items (e.g., “I feel depressed”). Responses are scored on a 4-point scale ranging from 0 (“completely disagree”) to 3 (“completely agree”), with total scores between 0 and 30. In this study, the Cronbach’s α coefficient for the SF-CES-D was 0.85.

#### 2.2.4 Creativity

We utilized the Runco Ideational Behavior Scale (RIBS), developed by Runco et al. (54), to assess creativity. This scale comprises 24 items, each scored on a 5-point Likert scale ranging from 1 (“never”) to 5 (“always”). The RIBS evaluates three dimensions of creativity: fluency (8 items, e.g., “I have some ways to make my work easier”), originality (9 items, e.g., “I have ideas that others might never have thought of”), and flexibility (7 items, e.g., “I consider various possible solutions when solving problems”). This scale has shown strong reliability and validity among Chinese adolescents (55). In our study, the Cronbach’s α coefficient for the RIBS was 0.85.

#### 2.2.5 Aggressive behavior

We used the Buss-Perry Aggression Questionnaire (BPAQ) to assess various facets of aggression. The BPAQ is widely recognized as a reliable instrument for measuring aggression in the Chinese population (56). It consists of four dimensions: physical aggression (5 items, e.g., “If I have to resort to force to protect my rights, I will”), verbal aggression (6 items, e.g., “When people disagree with my opinion, I openly tell them”), anger (3 items, e.g., “I flare up quickly but get over it quickly”), and hostility (8 items, e.g., “Some of my friends think I’m a hothead”). Each item is scored on a scale from 1 (“completely disagree”) to 5 (“completely agree”). Higher scores indicate greater aggression behavior. In this study, the Cronbach’s α coefficient for the BPAQ was 0.85.

#### 2.2.6 Data analysis

Data availability analysis: We assessed common method bias in the questionnaire data and performed a Hardy-Weinberg equilibrium test to evaluate the genetic sampling.

Calculation and analysis of MGPS: Following the theory of biological sensitivity to context (57) and previous research, we selected serotonin-system genes relevant to stress sensitivity and social adjustment. The candidate genes included TPH2 (rs4570625, rs4290270), SCL6A4 (rs1042173), HTR1A (rs1364043, rs6295), HTR2A (rs7997012, rs6311), and MAOA (rs6323). Alleles previously linked to heightened environmental sensitivity, emotional reactivity, or maladaptive adjustment were coded as putative sensitivity alleles and assigned a score of 1. MGPS were calculated by summing unweighted putative sensitivity alleles across loci. Higher MGPS values indicated greater genetic susceptibility to environmental influences. Detailed SNP coding procedures, allele classifications, and references are provided in **Appendix 1**.

Testing for correlations and G × E × E effects: We employed correlation analyses to examine the relationship between core variables and explored the G × E × E effects on adolescent social adjustment. All primary regression models controlled for demographic covariates, including gender, only-child status, family SES, and dummy-coded parental marital status. We further used coefficient-difference tests to formally compare the corresponding father-child and mother-child effects. To better understand these interactions, we generated simple slopes to investigate the specific moderating effects of serotonergic MGPS.

Validating gene-environment interaction models: To elucidate the theoretical model underlying gene-environment interactions, we utilized region of significance (RoS) analysis (58). This method assessed the impact of serotonergic MGPS and environmental factors (parent-child relationship and CA) on adolescent social adjustment, aiming to determine alignment with the diathesis-stress or differential susceptibility model. RoS analysis identifies optimal regions of significance within M ± 2 SD, covering 95% of the data, and quantifies model validity using the Proportion of Interaction Index (PoI) and Proportion Affected Index (PA). These indices help determine whether interactions fit the diathesis-stress model or the differential susceptibility model. Additionally, we examined cross-over points and tested the significance of X² (or ZX²) to confirm non-linear relationship and avoid misinterpreting interactions.

## 3 Results

### 3.1 Data availability analysis

We assessed common method bias in the questionnaire data using Harman’s single-factor test. The results revealed 12 factors with eigenvalues greater than 1, with the first factor explaining 20.32% of the variance, below the critical threshold of 40% (59). Additionally, a confirmatory factor analysis using Mplus was performed. The single-factor model showed poor fit: χ2 = 695.30, df = 135, CFI = 0.49, TLI = 0.42, RMSEA = 0.18, SRMR = 0.16. These findings suggest that common method bias is not a significant issue.

We also conducted Hardy-Weinberg equilibrium tests for all gene loci (see **Appendix 2**). The observed genotype frequencies were consistent with expected frequencies, adhering to *Hardy*-Weinberg equilibrium. Moreover, the minor allele frequencies (MAF) were above 0.05 and were comparable to the MAFs reported for the Beijing Han Chinese population in the 1000 Genomes Project (60), supporting the quality of our genetic sampling.

### 3.2 Descriptive statistics and correlation analysis

The serotonergic MGPS scores ranged from 2 to 14. **Table 1** provides the descriptive statistics and correlation analysis results for the core variables. The analysis revealed that the serotonergic MGPS was not correlated with any other variables. However, significant correlations were observed between parent-child relationship, CA, depressive symptoms, creativity, and aggressive behavior.

**Table 1:** Descriptive statistics and correlation analysis results.

Variables	1	2	3	4	5	6	7
FCR	1						
MCR	0.72**	1					
CA	−0.36**	−0.29**	1				
Depressive symptoms	−0.30**	−0.25**	0.27**	1			
Creativity	0.23**	0.23**	-0.05	−0.05	1		
Aggressive behavior	−0.26**	−0.25**	0.33**	0.31**	0.02	1	
Serotonergic MGPS	0.02	0.04	−0.03	-0.06	0.05	-0.02	1
M ± SD	30.04 ± 9.26	32.26 ± 8.42	0.93 ± 1.40	17.70 ± 6.01	9.70 ± 2.07	73.08 ± 18.90	6.97 ± 1.88
N	698	699	700	699	697	696	700
Cronbach’s α	0.92	0.92	0.72	0.85	0.85	0.85	_

1 for FCR, 2 for MCR, 3 for CA, 4 for depressive symptoms, 5 for creativity, 6 for aggressive behavior, and 7 for serotonergic MGPS. *p <.05, **p <.01, ***p <.001. These notations apply throughout. Bootstrap 95% confidence intervals for the Pearson correlations are provided in **Appendix 3**.

### 3.3 Testing the moderating role of serotonergic genetic variation on stress sensitivity and social adjustment

Using SPSS 27.0 PROCESS macro v3.4 (Model 3), we analyzed the interactions among the three factors. All continuous variables were standardized. Each model adjusted for gender, only-child status, parental marital status, and family SES as covariates and included all main effects and their interactions. All categorical covariates were appropriately dummy coded prior to regression analysis. The results are presented in **Tables 2** and **3**. Parent-child relationship and CA had effects on adolescent social adjustment (|βs_parent-child relationship_| > 0.15, *ps* <.001; |βs_CA_| > 0.18, *ps* <.001). However, CA was not associated to creativity.

**Table 2:** Father-child model: G × E × E effects test for adolescent social adjustment.

Predictors	Depression symptoms				Creativity				Aggressive behavior			
	β	t	p	95% CI	β	t	p	95% CI	β	t	p	95% CI
Gender	0.21	2.95**	**.01**	(0.07, 0.35)	0.04	0.52	.61	(-0.11, 0.19)	-0.002	-0.03	.97	(-0.14, 0.14)
Only-child Status	-0.01	-0.07	.94	(-0.14, 0.13)	-0.11	-1.46	.15	(-0.25, 0.04)	0.03	0.45	.65	(-0.11, 0.17)
Family SES	0.17	3.34***	**<.001**	(0.07, 0.27)	-0.07	-1.27	.21	(-0.17, 0.04)	0.05	1.05	.29	(-0.05, 0.15)
Marital 1	0.11	0.63	.53	(-0.24, 0.47)	-0.01	-0.03	.98	(-0.38, 0.36)	-0.01	-0.06	.95	(-0.36, 0.34)
Marital 2	0.35	2.48*	**.01**	(0.07, 0.63)	0.38	2.55*	**.01**	(0.09, 0.67)	0.02	0.12	.90	(-0.26, 0.30)
Marital 3	0.46	3.04**	**.003**	(0.16, 0.76)	0.03	0.18	.86	(-0.28, 0.34)	0.03	0.19	.85	(-0.27, 0.33)
FCR	-0.22	-5.80***	**<.001**	(-0.30, -0.15)	0.26	6.47***	**<.001**	(0.18, 0.34)	-0.15	-3.86***	**<.001**	(-0.22, -0.07)
CA	0.18	4.21***	**<.001**	(0.09, 0.26)	0.02	0.53	.60	(-0.06, 0.11)	0.35	8.43***	**<.001**	(0.27, 0.43)
MGPS	-0.04	-1.18	.24	(-0.12, 0.03)	0.05	1.22	.22	(-0.03, 0.12)	-0.04	-1.07	.29	(-0.11, 0.03)
FCR × CA	0.04	1.24	.22	(-0.02, 0.11)	-0.04	-1.09	.28	(-0.11, 0.03)	0.11	3.25**	**.001**	(0.04, 0.17)
FCR × G	0.01	0.39	.70	(-0.06, 0.09)	0.10	2.65**	**.008**	(0.03, 0.18)	-0.01	-0.20	.84	(-0.08, 0.07)
CA × G	-0.05	-1.16	.25	(-0.13, 0.03)	-0.01	-0.25	.80	(-0.10, 0.07)	-0.05	-1.32	.19	(-0.14, 0.03)
FCR × CA × G	-0.02	-0.59	.56	(-0.09, 0.05)	-0.03	-0.73	.46	(-0.10, 0.05)	-0.10	-2.66**	**.008**	(-0.17, -0.03)
R2	0.17				0.09				0.16			
F	10.81***				4.86***				10.16***			

G for serotonergic MGPS. Parental marital status was dummy-coded with first marriage as the reference group: Marital 1 = divorced parents, Marital 2 = remarried parents, and Marital 3 = other marital status. Significant results are highlighted in bold.

**Table 3:** Mother-child model: G × E × E effects test for adolescent social adjustment.

Predictors	Depression symptoms				Creativity				Aggressive behavior			
	β	t	p	95% CI	β	t	p	95% CI	β	t	p	95% CI
Gender	0.21	2.89**	**.01**	(0.07, 0.35)	0.04	0.50	.62	(-0.11, 0.19)	0.01	0.20	.84	(-0.13, 0.16)
Only-child Status	-0.02	-0.31	.76	(-0.16, 0.12)	-0.10	-1.40	.16	(-0.25, 0.04)	0.03	0.37	.71	(-0.11, 0.17)
Family SES	0.18	3.54***	**<.001**	(0.08, 0.28)	-0.07	-1.35	.18	(-0.18, 0.03)	0.06	1.19	.24	(-0.04, 0.16)
Marital 1	0.12	0.67	.51	(-0.23, 0.47)	0.02	0.12	.90	(-0.35, 0.39)	0.03	0.15	.88	(-0.33, 0.38)
Marital 2	0.39	2.79**	**.01**	(0.12, 0.67)	0.30	2.01*	.045	(0.01, 0.59)	0.01	0.07	.94	(-0.27, 0.29)
Marital 3	0.47	3.13**	**.00**	(0.18, 0.77)	0.06	0.38	.71	(-0.25, 0.37)	0.00	0.01	.99	(-0.30, 0.30)
MCR	-0.19	-5.12***	**<.001**	(-0.26, -0.12)	0.24	6.19***	**<.001**	(0.16, 0.32)	-0.16	-4.19***	**<.001**	(-0.23, -0.08)
CA	0.23	5.77***	**<.001**	(0.15, 0.30)	0.00	0.11	.91	(-0.08, 0.09)	0.33	8.45***	**<.001**	(0.26, 0.41)
MGPS	-0.06	-1.65	.10	(-0.13, 0.01)	0.05	1.20	.23	(-0.03, 0.12)	-0.03	-0.72	.47	(-0.10, 0.05)
MCR × CA	0.12	3.40***	**<.001**	(0.05, 0.18)	-0.04	-1.00	.318	(-0.11, 0.03)	0.10	2.85**	**.005**	(0.03, 0.17)
MCR × G	-0.03	-0.78	.44	(-0.10, 0.04)	0.13	3.38***	**<.001**	(0.06, 0.21)	0.02	0.53	.60	(-0.05, 0.09)
CA × G	-0.10	-2.35*	**.02**	(-0.18, -0.02)	0.01	0.33	.74	(-0.07, 0.10)	-0.03	-0.82	.41	(-0.11, 0.05)
MCR × CA × G	-0.08	-2.19*	**.03**	(-0.16, -0.01)	0.00	0.03	.97	(-0.08, 0.08)	-0.08	-1.99*	**.048**	(-0.15, -0.001)
R2	0.18				0.09				0.16			
F	11.47***				4.91***				9.84***			

G for serotonergic MGPS. Parental marital status was dummy-coded with first marriage as the reference group: Marital 1 = divorced parents, Marital 2 = remarried parents, and Marital 3 = other marital status. Significant results are highlighted in bold.

In the depressive symptoms model, the three-way interaction among MCR, CA, and MGPS was significant (β = -0.08, *p* = .03). Additionally, the interaction between MCR and CA was significant (β = 0.12, *p* <.001). In the creativity model, the interaction between parent-child relationship and serotonergic MGPS was significant (β_father-child_ = 0.10, *p* = .01; β_mother-child_ = 0.13, *p* <.001). Finally, in the aggressive behavior model, the three-way interactions significantly predicted adolescent aggressive behavior (β_father-child_ = -0.10, *p* = .01; β_mother-child_ = -0.08, *p* = .048). The interaction between parent-child relationship and CA affected adolescent aggression behavior (β_father-child_ = 0.11, p <.001; β_mother-child_ = 0.10, *p* = .005). The G × E × E effects accounted for approximately 1% of the variance in adolescent social adjustment (ΔR²_depressive symptoms_ = 0.01, *p* = .03; ΔR²_creativity_ = 0.001, *p* = .97; ΔR²_aggression behavior_ = 0.01, *p* = .01). Although statistically significant, these incremental effects were small in magnitude and should therefore be interpreted cautiously.

Additional coefficient-difference tests were conducted to formally compare the corresponding father-child and mother-child effects. The results showed that father-child and mother-child G × E × E effects differed significantly only for depressive symptoms (Δβ = 0.21, *p* = .036), but not for aggressive behavior (Δβ = -0.09, *p* = .397). Specifically, the G × E × E effect predicting depressive symptoms was more evident in the mother-child model. For creativity, the father-child and mother-child G × E effects also did not differ significantly (Δβ = -0.10, *p* = .347; see **Appendix 4**).

### 3.4 Simple slope analysis

**Figure 2:**
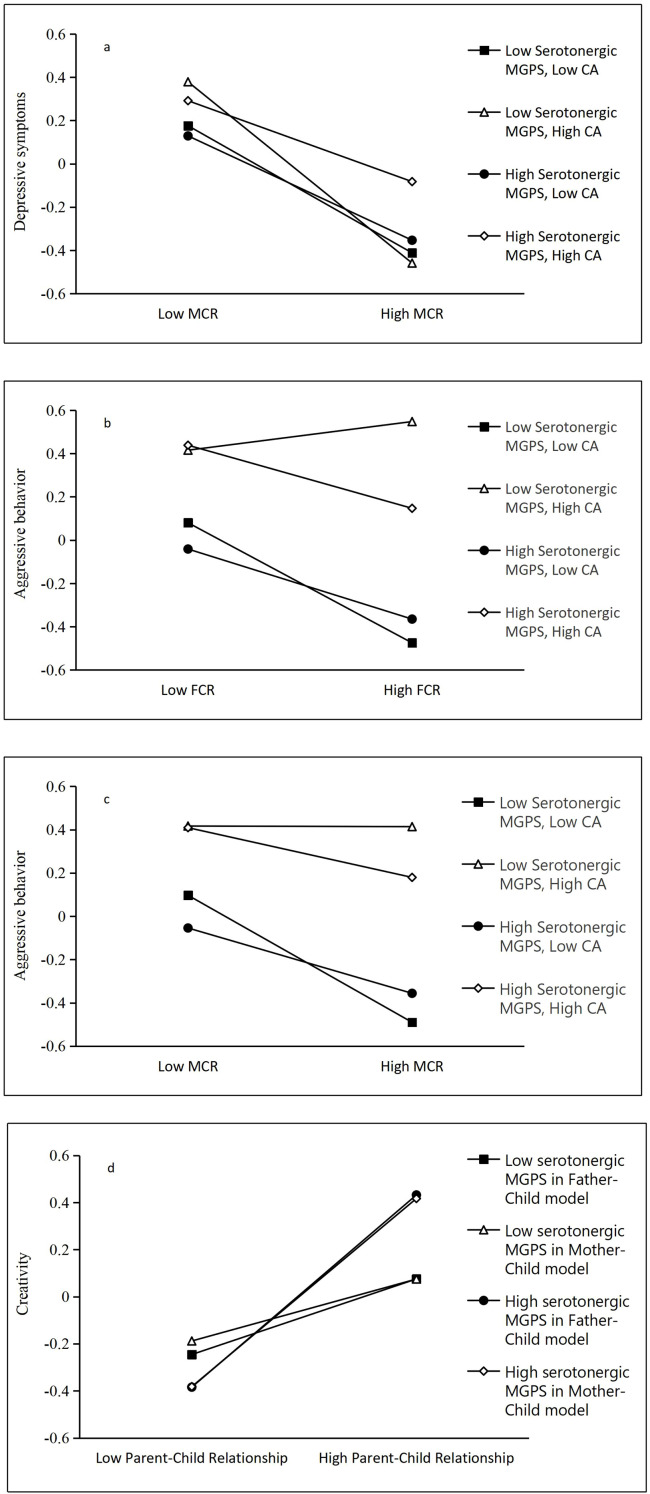
Simple slope plot. **(A)** Refers to the depressive symptoms in mother-child model, **(B)** refers to the aggressive behavior in father-child model, and **(C)** refers to the aggressive behavior in mother-child model, **(D)** refers to the creativity model.

**Figure 2A** illustrated that for adolescents with low serotonergic MGPS, the MCR was correlated with depressive symptoms in cases of low CA (β = -0.29, *p* <.001). However, in cases of high CA, the MCR did not affect depressive symptoms (β = 0.04, *p* = .56). For adolescents with high serotonergic MGPS, the MCR was correlated with depressive symptoms regardless of the level of CA (β_LCA_ = -0.24, *p* <.001; β_HCA_ = -0.19, *p* = .01). The Johnson-Neyman analysis indicated that the conditional MCR × CA interaction became non-significant when standardized serotonergic MGPS was greater than 0.45. To further explain this effect, we examined whether the genetic moderation of proximal stress exposure is intensified by distal stress exposure (i.e., another decomposition of the same G × E × E interaction, with E1 being CA and E2 being the parent-child relationship). The results show significant differences in the G × E interaction at different levels of CA (β_LCA_ = -0.03, *p* = .58; β_HCA_ = -0.11, *p* = .03).

**Figure 2B** illustrated that for adolescents with low serotonergic MGPS, the FCR was correlated with aggressive behavior under low CA (β = -0.28, *p* <.001). However, this relationship was not significant under high CA (β = 0.07, p = .35). In contrast, for adolescents with high serotonergic MGPS, the FCR was significantly associated with aggressive behavior under low CA (β_LCA_ = -0.16, p = .02), whereas this association did not reach conventional statistical significance under high CA (β_HCA_ = -0.15, p = .052). The Johnson-Neyman analysis indicated that the conditional FCR × CA interaction became non-significant when standardized serotonergic MGPS was greater than 0.39. To further understand this effect, we examined whether the genetic moderation of proximal stress exposure was intensified by distal stress exposure. The results showed significant differences in the G × E interaction at different levels of CA (β_LCA_ = 0.06, *p* = .20; β_HCA_ = -0.11, *p* = .045), similar to the previous findings.

**Figure 2C** illustrated that for adolescents with low serotonergic MGPS, the MCR was significantly correlated with aggressive behavior under low CA (β = -0.29, *p* <.001). However, this relationship was not significant under high CA (β = -0.001, *p* = .98). For adolescents with high serotonergic MGPS, the MCR was correlated with aggressive behavior only under low CA (β_LCA_ = -0.15, *p* = .03; β_HCA_ = -0.11, *p* = .12). The Johnson-Neyman analysis indicated that the conditional MCR × CA interaction became non-significant when standardized serotonergic MGPS was greater than 0.31. To further understand this effect, we examined whether the genetic moderation of proximal stress exposure was intensified by distal stress exposure. The results showed that the G × E interaction was not significant at different levels of CA (β_LCA_ = 0.07, *p* = .13; β_HCA_ = -0.06, *p* = .28).

Finally, although no G × E × E interaction was found in adolescent creativity, a two-way interaction between serotonergic MGPS and the parent-child relationship was identified. Therefore, we used PROCESS Model 1 to examine the moderating effect of serotonergic MGPS on the influence of the parent-child relationship on creativity. The interaction was significant (β_father-child_ = 0.11, *p* = .005; β_mother-child_ = 0.13, *p* <.001; specific regression coefficients are presented in the **Appendix 5**). Moreover, the G × E effect accounted for approximately 1%-2% of the variance in creativity (ΔR²_father-child_ = 0.01, *p* <.01; ΔR²_mother-child_ = 0.02, *p* <.001). Simple slope tests (**Figure 2D**) showed that, compared to adolescents with low serotonergic MGPS (β_father-child_ = 0.14, *p* = .006; β_mother-child_ = 0.10, *p* = .04), the influence of the parent-child relationship on adolescent creativity was greater among those with high serotonergic MGPS (β_father-child_ = 0.35, *p* <.001; β_mother-child_ = 0.37, *p* <.001). These results were generally consistent with the differential susceptibility model under varying environment. Notably, creativity may not follow the same stress-sensitization process as depressive symptoms and aggressive behavior, but may instead reflect sensitivity to current supportive relational contexts.

### 3.5 Validation of gene-environment interaction theoretical models

**Figure 3:**
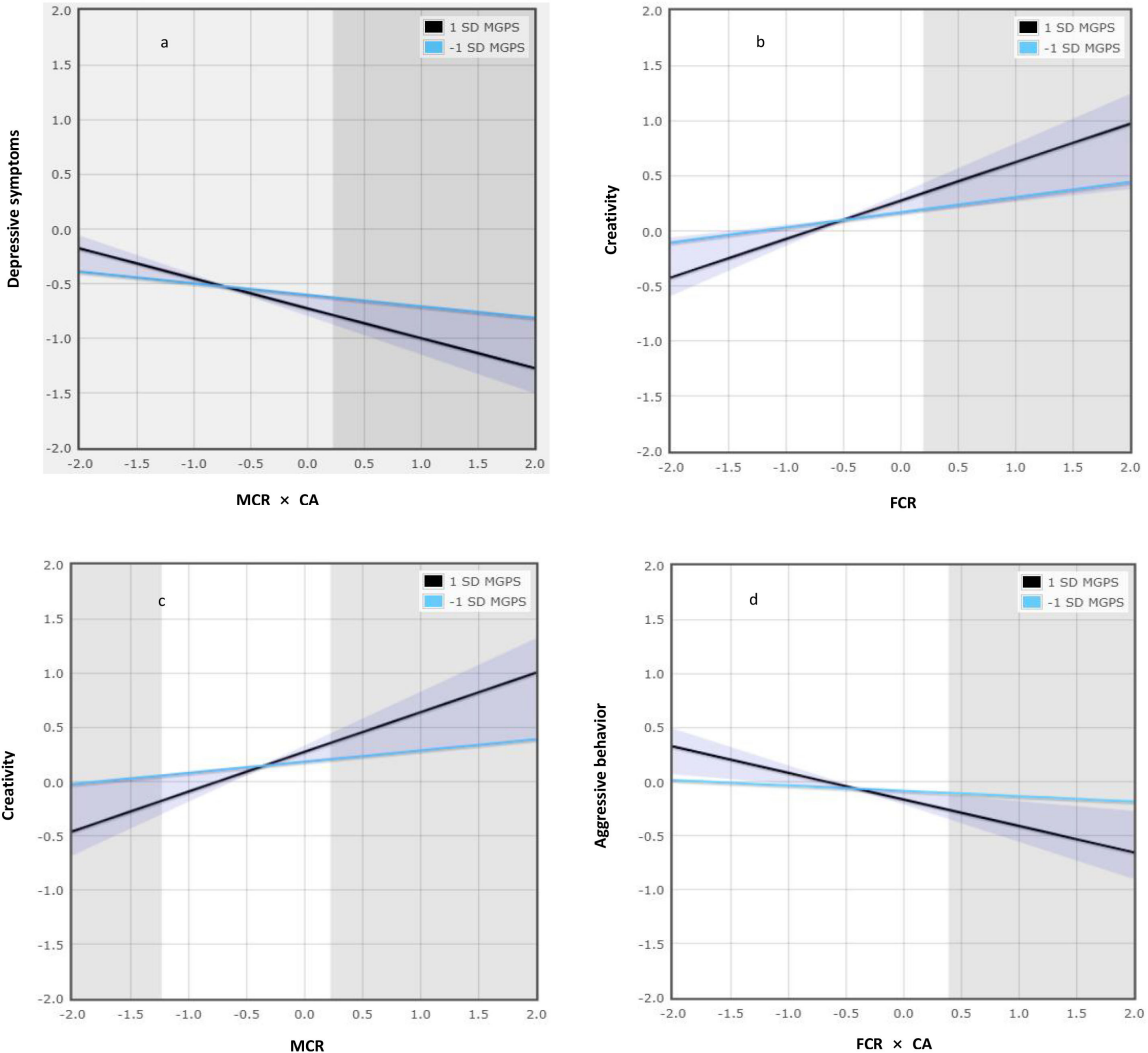
RoS test results for gene-environment interaction on social adjustment. **(a)** refers to depressive symptoms in the mother-child model, **(b)** refers to creativity in the father-child model, **(c)** refers to creativity in the mother-child model, and **(d)** refers to aggressive behavior in the father-child model.

We employed the region of significance (RoS) method to further investigate the interaction patterns between serotonergic MGPS and environmental factors (see **Table 4**, **Figure 3**). The results indicated that across the four equations, regardless of the type of environment or the psychological outcome variable, the Proportion of Interaction (PoI) index ranged from 0.65 to 0.84, and the Proportion Affected (PA) index ranged from 0.62 to 0.79, both approximating 0.5. Moreover, the X² and ZX² tests did not predict adolescent social adjustment, thus ruling out non-linear relationship. In summary, the interaction patterns between serotonergic MGPS and the environment were generally consistent with the differential susceptibility model. However, not all interaction patterns showed fully symmetrical benefit-and-risk effects.

**Table 4:** The results of gene-environment interaction RoS testing.

Outcomes	Predictors	Significant range		PoI	PA	X2/ZX2	Crossover point	Figure
		Lower threshold	Upper threshold					
Depressive symptoms	(MCR*CA) × MGPS	−3.82	-0.09	0.84	0.79	ns.	-0.73	3a
Creativity	FCR × MGPS	−1.25	0.21	0.67	0.63	ns.	-0.34	3b
Creativity	MCR × MGPS	-0.91	0.19	0.65	0.62	ns.	-0.31	3c
Aggressive behavior	(FCR*CA) × MGPS	-1.93	0.27	0.73	0.70	ns.	-0.46	3d

*ns.* means non-significant.

## 4 Discussion

Based on the stress sensitivity model and the emerging G × E × E model, this study incorporated key genetic factors (serotonergic MGPS), childhood adversity (CA), and proximal stress (parent-child relationship). The results provide preliminary evidence that genetic factors may moderate the stress sensitivity resulting from early adversity. Specifically, significant G × E × E interactions of serotonergic MGPS, CA, and parent-child relationships were observed for depressive symptoms and aggressive behavior. Creativity, by contrast, showed no significant three-way interaction but a G × E interaction instead. These findings may help enriching our understanding of the role of G × E in the development of psychopathology.

### 4.1 Evidence linking environment to long-term health outcomes

A substantial body of research has extensively examined the impact of CA on the risk of developing psychological disorders, with findings indicating that these changes may persist and elevate the risk of mental health problems throughout life (61, 62). Consistent with previous research, our study found that CA affected adolescent social adjustment. Furthermore, both animal and human neurobiological studies have shown that CA is associated with cellular aging, alterations in the serotonin, changes in related neurotransmitter systems, and can even impact brain structure, particularly the hippocampus, with lifelong effects (63). Our results also highlight the significant influence of parent-child relationship, a crucial interpersonal factor during adolescence, on social adjustment. Prior studies have demonstrated that high-quality parent-child relationship enhance academic performance, self-esteem, and a sense of belonging in adolescents, while also reducing the risk of psychological issues and disorders (64).

### 4.2 Gene-environment interaction on adolescent social adjustment

This study examined the moderating role of serotonergic MGPS in the interaction between CA and parent-child relationship on adolescent social adjustment. The results, supported by simple slope analysis and Johnson-Neyman tests, indicate that as serotonergic MGPS increases, the interaction between parent-child relationship and CA strengthens. This finding suggests that people with high genetic sensitivity exhibit greater stress sensitivity. Our findings are consistent with previous studies (34), which reported an interaction between CA and genetic sensitivity scores. These results offer potential explanations for the inconsistent findings in current G × E research.

First, previous literature has not thoroughly detailed how genetic sensitivity in adolescents moderates the link between parent-child relationship and social adjustment. However, a comprehensive review of serotonergic neurophysiological functions suggests plausible hypotheses. The serotonergic system is associated with reward and punishment, as well as sensation-seeking behaviors (e.g., 65, 66). Soga et al. (67) emphasized that the expression and genetic variations of the serotonergic system, particularly in the amygdala, hippocampus, and dorsal raphe nucleus, are closely related to social adjustment. Therefore, adolescents with high genetic sensitivity might be more affected by environmental factors, such as parental warmth and support, or parental hostility and anger, compared to their peers. Another potential mechanism could involve DNA methylation and changes in gene expression triggered by environmental stimuli. For instance, in a systematic review of 72 studies, Cecil et al. (68) found that, despite some inconsistent findings, there is overall support for an association between CA and DNA methylation.

Second, significant G × E × E interactions were only observed for depressive symptoms and aggressive behavior, both of which represent stress-related maladjustment outcomes (69, 70). One possible explanation is that these outcomes are more directly tied to emotional reactivity and stress sensitization processes (42). Adolescents with higher serotonergic genetic sensitivity may be more reactive to current interpersonal environments following CA, thereby increasing vulnerability to emotional and behavioral dysregulation (42, 71). In contrast, creativity showed only G × E interactions, with no significant G × E × E effect. Creativity may rely more heavily on current relational support and environmental enrichment than on stress sensitization linked to CA (72). Positive parent-child relationship can create emotionally supportive and cognitively stimulating environments that facilitate the expression of genetic plasticity in creative functioning (72, 73). Accordingly, creativity appears particularly susceptible to the interplay between current family relationship and genetic susceptibility, yet less influenced by the combined effects of CA and present parent-child relationship. In addition, creativity was not significantly correlated with depressive symptoms or aggressive behavior, suggesting that it may represent a distinct positive adjustment domain rather than merely the absence of maladjustment (4, 74).

Third, formal coefficient-difference tests showed a significant father-child versus mother-child difference only for depressive symptoms, with the G × E × E effect being more evident in the mother-child model. This pattern suggests that the joint influence of serotonergic MGPS, childhood abuse, and parent-child relationship on depressive symptoms may be more salient in the mother-child context. This may be because depressive symptoms are closely related to emotional security, perceived support, and daily emotional communication (75). In many Chinese families, mothers are often more involved in daily caregiving and emotional interaction with adolescents (76). In contrast, aggressive behavior and creativity may be shaped by broader family interaction patterns involving both parents (77, 78).

Finally, it is important to note that many prior G × E studies have primarily focused on negative environments or adversities (79, 80). These studies often assume that the absence of negative environments equals positive environments (e.g., low parental warmth instead of hostility) or that low negative psychological outcomes signify high levels of mental health (e.g., low depressive symptoms as well-being; see 81). Our study examines the interactions of parent-child relationship, CA, and serotonergic MGPS on social adjustment, including depressive symptoms, creativity, and aggressive behavior. Both simple slope analyses and region-of-significance analyses were broadly consistent with the differential susceptibility framework (82). Moreover, robustness tests reveal that G × E × E interactions remain significant in all analyses, even when covariate interactions are included. This finding indicates that G × E effects are not driven by confounding factors but by specific genetic and environmental variables (see 83). Behavioral geneticists emphasize the importance of excluding gene-environment correlations when interpreting G × E effects (84). Correlation analyses show that serotonergic MGPS is not associated with psychosocial variables.

### 4.3 Limitations and future directions

This study has several limitations that warrant careful consideration. First, although the sample size provided adequate statistical power, all participants were drawn from a single region, which may limit the generalizability of our findings. Second, although we employed a longitudinal design, relying on adolescents’ retrospective accounts of CA introduces recall bias and measurement error. The possibility of reverse causality cannot be entirely ruled out; adolescents with more depressive symptoms might be more likely to recall adverse childhood experiences (85). Future studies utilizing prospective designs that span childhood, and adolescence would better establish the temporal sequence of these variables. Third, although the hypotheses and analytic models were theory-driven, the use of two parent-child models, three outcomes, multiple interaction terms, simple slope analyses, and RoS analyses may have increased the likelihood of Type I error (86). Therefore, significant effects, especially those with small effect sizes or p values close to.05, should be interpreted cautiously and replicated in independent samples with appropriate multiple-testing control procedures, such as false discovery rate correction (87). Forth, although the MGPS approach may improve statistical power relative to single candidate-gene analyses, candidate-gene-based MGPS approaches remain debated in psychiatric genetics (88). This approach relies on existing candidate gene research databases that often yield inconsistent results for specific alleles under different conditions (14). Moreover, the selected SNPs were based on biological relevance to the serotonin system and prior candidate-gene literature rather than established genome-wide significant evidence for adolescent social adjustment (14, 88). The unweighted additive score also assumes equal contribution of coded alleles across loci (14). Future studies employing genome-wide polygenic scores and independent replication samples may provide more robust evidence regarding the associations between genetic sensitivity and adolescent social adjustment (89, 90). Finally, although we discussed several possible biological pathways, including serotonergic regulation, epigenetic processes, and neural responses in stress-related brain regions, these mechanisms were not directly measured in the present study. Therefore, these biological explanations should be interpreted cautiously. Future studies should incorporate epigenetic, neuroendocrine, and neuroimaging measures to directly examine the proposed mechanisms.

## 5 Final remark

This study provides preliminary evidence that serotonergic multilocus genetic variation may moderate adolescent stress sensitivity. Specifically, serotonergic MGPS interacted with CA and parent-child relationship in predicting social adjustment. Significant gene × environment × environment (G × E × E) interactions were observed for depressive symptoms and aggressive behavior. In contrast, creativity was associated with a significant gene × environment (G × E) interaction between serotonergic MGPS and parent-child relationship, whereas no significant G × E × E interaction was found. These results suggest that future research should further investigate individual differences in environmental sensitivity and explore whether supportive parent-child relationships may promote adolescent social adjustment, particularly among youth exposed to early adversity (91).

## Data Availability

The raw data supporting the conclusions of this article will be made available by the authors, without undue reservation.
