# Supplementary material for: The impact of parent-child relationship on adolescent social adjustment following childhood abuse: the moderation of the serotonergic multilocus genetic variation
Source: Front Psychiatry. 2026 Aug 4;17:1887601. doi: 10.3389/fpsyt.2026.1887601 (PMC13481699; doi:10.3389/fpsyt.2026.1887601)
Supplement: Supplementary file 1 [file DataSheet1.docx]

**Appendix 1**

The figure below illustrates the cooperative action of serotonin within the synapse. It details the mechanisms of serotonin synthesis, release, action, and reuptake in neural transmission. Understanding this process is crucial for comprehending serotonin's role in regulating mood, cognitive functions, sleep, and more. The specific steps are as follows:

**1. Synthesis:** The synthesis of serotonin begins with tryptophan, an essential amino acid, which is converted into the serotonin precursor 5-hydroxytryptophan (5-HTP) by the enzyme tryptophan hydroxylase. 5-HTP is then further converted into serotonin.

**2. Storage and Release:** Once synthesized, serotonin is stored in vesicles within the presynaptic neuron. When the neuron is activated, these vesicles release serotonin into the synaptic cleft.

3. Action: **Postsynaptic Action:** Serotonin released into the synaptic cleft can bind to receptors located on the postsynaptic neuron (e.g., 5-HT2A), triggering signaling pathways that influence various physiological functions. **Autoinhibition:** Serotonin can also bind to 5-HT1A receptors, providing feedback inhibition.

4. Reuptake and Degradation: **Reuptake:** Serotonin in the synaptic cleft is reabsorbed into the presynaptic neuron through the serotonin transporter (SERT), which allows serotonin to be recycled. **Degradation:** Serotonin that is not reabsorbed can be broken down by monoamine oxidase (MAO), with the resulting metabolites excreted in the urine.

Based on this genetic approach, the candidate genes selected for this study include:

**1. TPH2 (Tryptophan Hydroxylase 2):** This is the rate-limiting enzyme that converts tryptophan into 5-HTP, playing a critical role in serotonin synthesis.

**2. SLC6A4 (Serotonin Transporter, 5-HTT):** This gene encodes SERT, which is crucial for the reuptake of serotonin, and is essential for regulating serotonin concentration in the synaptic cleft.

**3. 5-HTR1A/2A (Serotonin 1A/2A Receptor Genes):** These receptor genes are involved in serotonin signaling, influencing various physiological and psychological processes.

**4. MAOA (Monoamine Oxidase A):** This enzyme is responsible for the metabolism of serotonin, affecting its concentration and activity in the body. Studying these genes can provide a deeper understanding of the role of the serotonin system in the onset and progression of depression, thereby offering a scientific basis for developing new therapeutic approaches.

***TPH2:*** Tryptophan (TRP) is converted into 5-hydroxytryptophan (5-HTP) by the enzyme tryptophan hydroxylase (TPH), and subsequently, 5-HTP is converted into serotonin by aromatic amino acid decarboxylase. This indicates that TPH, as the rate-limiting enzyme in serotonin synthesis, directly determines the rate of serotonin production. Research has revealed the presence of two isoforms of TPH in humans and other mammals, namely TPH1 and TPH2. These isoforms share 71% similarity in their amino acid sequences, but TPH2 has 44 unique amino acids at its N-terminus that are only present in TPH2. Animal model studies have shown that the mRNA level of TPH2 in the mouse brainstem is approximately 150 times higher than that of TPH1, suggesting that TPH2 plays a more critical role in serotonin synthesis in the brain (Schuhmacher et al., 2015). Therefore, TPH2's impact on mental health disorders is particularly significant, and its study is crucial for understanding and treating these conditions.

***rs4570625:*** In a study examining the association between TPH1 and TPH2 gene single nucleotide polymorphisms and depression among 280 Polish patients with major depressive disorder and 230 healthy volunteers, results indicated that the G/G homozygous genotype and the G allele at the rs4570625 locus were associated with depression (Wigner et al., 2018). Moreover, Chi et al. (2013) studied associations of the above SNP (rs4570625 and rs4565946) with anxiety disorders in 112 adult patients with poststroke anxiety and 246 healthy vol- unteers in the Chinese population and demonstrated a sta- tistically significant increase in the risk of developing post- stroke anxiety with carriership of the G allele of the SNP rs4570625 (OR = 1.374, 95% CI = 1.001–1.887, p = 0.049). In accordance with Vrshek-Schallhorn et al. (2015), TPH2 rs4570625 G-allele and THR1A rs6295 G-allele are risk alleles that affect serotonin function

Chi, S., Teng, L., Song, J. H., Zhou, C., Pan, W. H., Zhao, R. L., & Zhang, C. (2013). Tryptophan hydroxylase 2 gene polymorphisms and poststroke anxiety disorders. *Journal of affective disorders, 144*(1-2), 179-182.

Vrshek-Schallhorn, S., Stroud, C. B., Mineka, S., Hammen, C., Zinbarg, R. E., Wolitzky-Taylor, K., & Craske, M. G. (2015). Chronic and episodic interpersonal stress as statistically unique predictors of depression in two samples of emerging adults. *Journal of abnormal psychology, 124*(4), 918.

Wigner, P., Czarny, P., Synowiec, E., Bijak, M., Białek, K., Talarowska, M., ... & Sliwinski, T. (2018). Association between single nucleotide polymorphisms of TPH1 and TPH2 genes, and depressive disorders. *Journal of cellular and Molecular Medicine, 22*(3), 1778-1791.

**rs4290270:** In a meta-analysis, Liu et al. (2022) highlighted the T allele of rs4290270 as a notable susceptibility factor for depression. Further, a study by Karanović et al. (2017) demonstrated that individuals with the TT homozygous genotype for rs4290270 who have experienced non-specific trauma are at the greatest risk for suicide attempts. This suggests that the rs4290270 polymorphism may interact with environmental stressors to significantly increase the likelihood of severe depressive symptoms and suicidal behavior.

Karanović, J., Ivković, M., Jovanović, V. M., Šviković, S., Pantović-Stefanović, M., Brkušanin, M., ... & Savić-Pavićević, D. (2017). Effect of childhood general traumas on suicide attempt depends on TPH2 and ADARB1 variants in psychiatric patients. *Journal of neural transmission, 124*, 621-629.

Liu, Z. L., Wang, X. Q., Liu, M. F., & Ye, B. J. (2022). Meta-analysis of association between TPH2 single nucleotide poiymorphism and depression. *Neuroscience & Biobehavioral Reviews, 134*, 104517.

***SCL6A4:*** The dynamic balance of serotonin is primarily maintained through the serotonin transporter (5-HTT), which reabsorbs serotonin from the synaptic cleft back into the presynaptic neurons. Abnormalities in this process are closely linked to the development of various psychiatric disorders. Therefore, the SLC6A4 gene, which encodes 5-HTT, has become a significant focus in psychiatric research. The SLC6A4 gene encodes a transmembrane transport protein composed of approximately 630 amino acid residues. It is located in the 17p11.1-17p12 region of human chromosomes, contains 14 exons, spans 37.8 kb in length, and includes up to 30 genetic variation sites. These sites include single nucleotide polymorphisms (SNPs), variable number tandem repeats (VNTR), and the 5-HTT gene-linked polymorphic region (5-HTTLPR).

***rs1042173:*** In a study investigating the relationship between serotonin pathway genes and smoking cessation among rural Chinese men, results showed that the T allele of rs1042173 had a negative impact on quitting smoking. Specifically, individuals carrying the rs1042173 T allele were less likely to quit smoking compared to those who did not carry the T allele (Wang et al., 2018). In a study examining the link between alcohol craving and consumption tendencies and the SLC6A4 gene, Ait-Daoud et al. (2012) found that individuals with the rs1042173 TT genotype of the SLC6A4 gene exhibited significantly higher levels of alcohol urge and craving when exposed to alcohol-related cues. Finally, in a study investigating psychomotor agitation and emotional instability in Brazilian patients with autism spectrum disorder, a nominal association was found among male patients between the rs1042173 GG genotype and reduced psychomotor agitation, compared to carriers of the T allele (Schuch et al., 2016).

Ait-Daoud, N., Seneviratne, C., Smith, J. B., Roache, J. D., Dawes, M. A., Liu, L., ... & Johnson, B. A. (2012). Preliminary evidence for cue-induced alcohol craving modulated by serotonin transporter gene polymorphism rs1042173. *Frontiers in psychiatry, 3*, 6.

Schuch, J. B., Müller, D., Endres, R. G., Bosa, C. A., Longo, D., Schuler-Faccini, L., ... & Roman, T. (2016). Psychomotor agitation and mood instability in patients with autism spectrum disorders: A possible effect of SLC6A4 gene?. *Research in Autism Spectrum Disorders, 26*, 48-56.

Wang, Q., Li, S., Li, H., & Jia, C. (2018). Association of serotonergic pathway genes with smoking cessation in a Chinese rural male population. *Addictive Behaviors, 80*, 34-38.

***The 5-HTR1A/2A gene:*** as one of the important candidate genes for depression, plays a critical role in the pathogenesis of depression. The human 5-HTR1A gene is located in the q12.3 region of chromosome 5 and encodes the 5-HTR1A receptor. When this receptor is activated, it can reduce the synthesis and release of serotonin in the central nervous system, primarily by inhibiting the activity of neurons in the raphe nuclei. Numerous psychopharmacological and neurobehavioral studies have found that abnormalities in serotonin function in the central nervous system are closely related to the onset of depression (Xue Kunxi, 2010). Overexpression or abnormal activity of the 5-HTR1A receptor is associated with the pathological state in the brains of depression patients and those who have died by suicide due to depression.

***THR1A:*** The 5-HT1A receptor has dual functions: on one hand, it acts as an autoreceptor on dendrites regulating serotonin synthesis; on the other hand, it functions as a postsynaptic receptor involved in influencing various brain regions modulated by serotonergic neurotransmission. Studies have found that the levels of 5-HT1A receptors in the brains of patients with major depressive disorder are elevated, a finding confirmed by postmortem and positron emission tomography (PET) imaging studies (Nautiyal & Hen, 2017). Furthermore, research by Donaldson et al. (2016) using gene association analysis pointed out the link between the 5-HT1A polymorphism rs6295 and brain receptor levels, further confirming its important role in the pathogenesis of depression. Additionally, studies have explored the interaction effects of the polymorphism at the rs6295 locus in the 5-HTR1A gene and parenting styles on the development of depression in adolescents, revealing a complex interaction between genetic and environmental factors (Wang Meiping et al., 2015). This suggests that, under specific environmental conditions, an individual's genetic background may influence their susceptibility to depression.

***rs1364043:*** Takekita et al. (2015) found a significant correlation between the rs1364043 T allele and the percent change in the PANSS 5-factor negative score. Additionally, in a genotyping study of five SNPs (single nucleotide polymorphisms) (rs1364043, rs10042486, rs6313, rs6311, rs17289304) from these genes among 752 SZ patients, 568 MDD patients, and 846 Chinese Han normal controls, results showed that the haplotype (CT) consisting of rs10042486 and rs1364043 exhibited a significant difference between SZ cases and healthy controls.

Takekita, Y., Fabbri, C., Kato, M., Nonen, S., Sakai, S., Sunada, N., ... & Serretti, A. (2015). HTR1A gene polymorphisms and 5-HT1A receptor partial agonist antipsychotics efficacy in schizophrenia. *Journal of clinical psychopharmacology, 35*(3), 220-227.

Zhang, R., Bi, Y., Niu, W., Huang, X., Chen, S., Li, X., ... & He, G. (2016). Association study of 5-HT1A, 5-HT2A polymorphisms with schizophrenia and major depressive disorder in the Han Chinese population. *Neuroscience Letters, 635*, 39-43.

***rs6295:*** In accordance with Vrshek-Schallhorn et al. (2015), TPH2 rs4570625 G-allele and THR1A rs6295 G-allele are risk alleles that affect serotonin function. Additionally, in a study involving a sample of Ukrainian suicide attempters, results indicated that the HTR1A gene rs6295 polymorphism G allele may play a role in suicide behavior associated with exposure to highly traumatic and/or stressful life events (Wasserman et al., 2006). Donaldson et al. (2016) genotyped a cohort of 1,412 individuals, which was randomly divided into discovery and replication cohorts, to investigate the association between rs6295 and five psychiatric outcomes: history of psychiatric hospitalization, history of suicide attempts, history of substance or alcohol abuse, current posttraumatic stress disorder (PTSD), and current depression. Our findings indicate that the rs6295 G allele is linked to an increased risk of substance abuse, psychiatric hospitalization, and suicide attempts.

Donaldson, Z. R., Le Francois, B., Santos, T. L., Almli, L. M., Boldrini, M., Champagne, F. A., ... & Hen, R. (2016). The functional serotonin 1a receptor promoter polymorphism, rs6295, is associated with psychiatric illness and differences in transcription. *Translational psychiatry, 6*(3), e746-e746.

Vrshek-Schallhorn, S., Stroud, C. B., Mineka, S., Hammen, C., Zinbarg, R. E., Wolitzky-Taylor, K., & Craske, M. G. (2015). Chronic and episodic interpersonal stress as statistically unique predictors of depression in two samples of emerging adults. *Journal of Abnormal Psychology, 124*, 918.

Wasserman, D., Geijer, T., Sokolowski, M., Rozanov, V., & Wasserman, J. (2006). The serotonin 1A receptor C (-1019) G polymorphism in relation to suicide attempt. *Behavioral and Brain Functions, 2*, 1-5.

***THR2A:*** The 5-HT2A receptors are widely distributed throughout the central nervous system, particularly in regions crucial for learning and cognition. These receptors play roles in key physiological processes such as memory, sleep, pain perception, eating behavior, and reward mechanisms. They are also closely associated with the pathogenesis of various neuropsychiatric disorders, including schizophrenia, depression, and anxiety disorders (Guiard & Giovanni, 2015). Research indicates that antagonists targeting the 5-HT2A receptors may be effective in combating mental illnesses and depression, while agonist-type ligands could enhance cognitive abilities and induce hallucinations. Additionally, genetic association studies have found that single nucleotide polymorphisms (SNPs) in the 5-HT2A receptor gene, such as rs6311, rs6313, and rs7997012, have significant correlations with the response of major depressive disorder (MDD) patients to antidepressant treatment. A review on the genetic mechanisms of serotonin receptors and depression highlighted a significant association between the A allele or A/A genotype of the 5-HT2A gene rs6311 polymorphism and depression.

***rs7997012:*** The A carriers (A/A+A/G) showed significantly higher rate of suicide attempt compared to the G/G when there is a history of child abuse (Shinozaki et al., 2013). In a study involving 46 adolescent patients treated with fluoxetine for major depressive disorder, results showed that after Bonferroni correction, the HTR2A rs7997012 SNP was significantly associated with clinical improvement. Specifically, individuals homozygous for the major allele (GG) exhibited the highest remission rates and the greatest reduction in scores on the Clinical Global Impressions-Severity (CGI-S) scale (Gassó et al., 2018). Finally, in evaluating the treatment response of 156 patients participating in an open-label clinical trial of venlafaxine XR for GAD over six months, Lohoff et al. (2013) found that individuals with the HTR2A rs7997012 single nucleotide polymorphism G allele showed better treatment outcomes over time.

Gassó, P., Blázquez, A., Rodríguez, N., Boloc, D., Torres, T., Mas, S., ... & Lázaro, L. (2018). Further support for the involvement of genetic variants related to the serotonergic pathway in the antidepressant response in children and adolescents after a 12-month follow-up: impact of the HTR2A rs7997012 polymorphism. *Journal of child and adolescent psychopharmacology, 28*(10), 711-718.

Lohoff, F. W., Aquino, T. D., Narasimhan, S., Multani, P. K., Etemad, B., & Rickels, K. (2013). Serotonin receptor 2A (HTR2A) gene polymorphism predicts treatment response to venlafaxine XR in generalized anxiety disorder. *The pharmacogenomics journal, 13*(1), 21-26.

Shinozaki, G., Romanowicz, M., Mrazek, D. A., & Kung, S. (2013). HTR2A gene–child abuse interaction and association with a history of suicide attempt among Caucasian depressed psychiatric inpatients. *Journal of affective disorders, 150*(3), 1200-1203.

***rs6311:*** The G allele of rs6311 is related to worse mental health and higher [alexithymia](https://www.sciencedirect.com/topics/medicine-and-dentistry/alexithymia) as compared to the AA genotype (Li et al., 2020). Two separate studies also found that the G allele of rs6311 is associated with a higher likelihood of developing depression (Choi et al., 2004; Noskova et al., 2009).

Choi, M.J., Lee, H.J., Lee, H.J., Ham B.J., Cha, J.H., Ryu, S.H. and Lee M.S. (2004) Association between Major Depressive Disorder and the -1438A/G Polymorphism of the Serotonin 2A Receptor Gene. *Neuropsychobiology, 49*, 38-41.
https://doi.org/10.1159/000075337

Li, X., He, L., Liu, J., Guo, W., Wang, Q., Fang, P., ... & Gong, P. (2020). The rs6311 of serotonin receptor 2A (5-HT2A) gene is associated with alexithymia and mental health. *Journal of affective disorders, 272*, 277-282.

Noskova, T. G., Kazantseva, A. V., Gareeva, A. E., Gaisyna, D. A., Tuktarova, S. U., & Khusnutdinova, E. K. (2009). Association of several polymorphic loci of serotoninergic genes with unipolar depression. *Russian journal of genetics, 45*, 742-748.

***MAOA gene:*** located in the X chromosome region p11.23-11.4, encodes monoamine oxidase A, a key enzyme that primarily acts in central nervous system regions such as the prefrontal cortex, hippocampus, and amygdala, and is closely related to human emotions and behavior (Buckholtz et al., 2008; Buckholtz & Meyer-Lindenberg, 2008). Therefore, MAOA gene polymorphisms are significant for understanding human emotion regulation and behavioral expression. The initial evidence linking the MAOA gene to human social behavior came from a study of a criminal family in the Netherlands (Saito et al., 2002). This study found that several male members of the family exhibited extreme antisocial behaviors, such as arson, robbery, and attempted rape, which were associated with specific mutations in their MAOA gene.

***rs6323:*** Maternal supportive parenting significantly negatively predicted depression in female adolescents with the GG genotype, but its predictive effect on depression in female adolescents with the TT genotype was not significant (Cao et al., 2016).

Cao, C., Wang, M., Ji, L., Wei, X., Cao, Y., & Zhang, W. (2016). The MAOA rs6323 polymorphism interacts with maternal supportive parenting in predicting adolescent depression: Testing the diathesis-stress and differential susceptibility hypotheses. *Acta Psychologica Sinica, 48*(1), 22.

**Appendix 2**

*SNP Data*

| Gene | Locus | Allele | MAF | MAF(1000g-CHBS) | Genotypes and Distribution | | | Hardy-Weinberg equilibrium | |
| --- | --- | --- | --- | --- | --- | --- | --- | --- | --- |
|  |  |  |  |  | 11 | 12 | 22 | *χ^2^* | *p* |
| *TPH2* | rs4570625 | T > G | 0.44 | 0.47 | 228（32.6%） | 328 （46.9%） | 144 （20.6%） | 1.69 | .43 |
|  | rs4290270 | A > T | 0.44 | 0.49 | 226 （32.3%） | 333 （47.6%） | 141 （20.1%） | 0.83 | .66 |
| *SCL6A4* | rs1042173 | C > A | 0.08 | 0.21 | 439 （62.7%） | 231 （33.0%） | 30 （4.3%） | 0.10 | .95 |
| *HTR1A* | rs1364043 | G > T | 0.39 | 0.39 | 264 （37.7%） | 329 （47.0%） | 107 （15.3%） | 0.10 | .95 |
|  | rs6295 | G > C | 0.24 | 0.24 | 407 （58.1%） | 257 （36.7%） | 36 （5.1%） | 0.64 | .73 |
| *HTR2A* | rs7997012 | G > A | 0.27 | 0.28 | 384 （54.9%） | 261 （37.3%） | 55 （7.9%） | 1.44 | .48 |
|  | rs6311 | T > C | 0.39 | 0.41 | 269 （38.4%） | 317 （45.3%） | 114 （16.3%） | 1.58 | .45 |
| *MAOA* | rs6323 | G > T | 0.41 | 0.38 | 332 （47.4%） | 163 （23.3%） | 205 （29.3%） | - | - |

*Note.* 11 = Frequency of homozygotes composed of the major allele. 12 = Heterozygote frequency. 22 = Frequency of homozygotes composed of the minor allele.

**Appendix 3**

*Pearson Correlations with Bootstrap 95% Confidence Intervals*

| Variables | 1 | 2 | 3 | 4 | 5 | 6 | 7 |
| --- | --- | --- | --- | --- | --- | --- | --- |
| Father-child relationship | — |  |  |  |  |  |  |
| Mother-child relationship | [0.648, 0.780] | — |  |  |  |  |  |
| Childhood abuse | [-0.432, -0.286] | [-0.367, -0.220] | — |  |  |  |  |
| Depressive symptoms | [-0.371, -0.229] | [-0.323, -0.175] | [0.198, 0.349] | — |  |  |  |
| Creativity | [0.156, 0.308] | [0.157, 0.308] | [-0.120, 0.030] | [-0.128, 0.026] | — |  |  |
| Aggressive behavior | [-0.335, -0.173] | [-0.327, -0.163] | [0.265, 0.399] | [0.241, 0.377] | [-0.069, 0.112] | — |  |
| Serotonergic MGPS | [-0.051, 0.096] | [-0.031, 0.113] | [-0.103, 0.039] | [-0.131, 0.018] | [-0.029, 0.120] | [-0.088, 0.056] | — |

*Note.* Values are bootstrap 95% confidence intervals for the Pearson correlation coefficients reported in Table 1. 1 for father-child relationship, 2 for mother-child relationship, 3 for childhood abuse, 4 for depressive symptoms, 5 for creativity, 6 for aggressive behavior, and 7 for serotonergic MGPS. **p* < .05, ***p* < .01, ****p* < .001.

**Appendix 4**

**Coefficient-Difference Tests Between Father-Child and Mother-Child Models**

| Outcome | Effect compared | Δβ | *t* | *p* | 95% CI |
| --- | --- | --- | --- | --- | --- |
| Depressive symptoms | FCR × CA × MGPS vs. MCR × CA × MGPS | 0.21 | 2.10* | .036 | (0.01, 0.41) |
| Creativity | FCR × MGPS vs. MCR × MGPS | -0.10 | -0.94 | .347 | (-0.30, 0.11) |
| Aggressive behavior | FCR × CA × MGPS vs. MCR × CA × MGPS | -0.09 | -0.85 | .397 | (-0.28, 0.11) |

Note. FCR = father-child relationship; MCR = mother-child relationship; CA = childhood abuse; MGPS = multilocus genetic profile score. Δβ represents the father-child coefficient minus the mother-child coefficient. Difference tests used reparameterized regression models with PC_Avg = (FCR + MCR)/2 and PC_Diff = (FCR − MCR)/2. All models controlled for gender, only-child status, family SES, and dummy-coded parental marital status. **p* < .05.

**Appendix 5**

**The Effects of Serotonergic MGPS and Parent-Child Relationships on Adolescents' Creativity**

| Predictors | creativity | | | |
| --- | --- | --- | --- | --- |
|  | β | *t* | *p* | 95% CI |
| Father-child relationship | 0.24 | **6.52^***^** | **< .001** | (.17, .32) |
| MGPS | 0.05 | 1.43 | .15 | (-.02, .13) |
| G * E | 0.11 | **2.82**** | **.005** | (.03, .18) |
| R^2^ | 0.08 | | | |
| F | 6.76^***^ | | | |

| Predictors | creativity | | | |
| --- | --- | --- | --- | --- |
|  | β | *t* | *p* | 95% CI |
| Mother-child relationship | 0.23 | **6.33^***^** | **< .001** | (.16, .31) |
| MGPS | 0.04 | 1.21 | .23 | (-.03, .12) |
| G * E | 0.13 | **3.49**** | **< .01** | (.06, .20) |
| R^2^ | 0.08 | | | |
| F | 6.98^***^ | | | |

**Appendix 6**

**The Effects of Single Gene-Environment Interactions on Adolescent Social adjustment**

***Father-child Model***

| Predictors | Depression symptoms | | Creativity | | Aggressive behavior | |
| --- | --- | --- | --- | --- | --- | --- |
|  | β | *p* | β | *p* | β | *p* |
| rs4570625 | -0.0001 | .99 | -0.06 | .21 | **-0.10*** | **.03** |
| rs4290270 | -0.04 | .47 | -0.02 | .71 | -0.02 | .68 |
| rs1042173 | **0.13*** | **.02** | -0.03 | .66 | -0.07 | .21 |
| rs1364043 | -0.07 | .14 | 0.08 | .11 | **-0.13**** | **.01** |
| rs6295 | 0.04 | .52 | -0.11 | .06 | 0.09 | .12 |
| rs7997012 | -0.08 | .19 | 0.07 | .29 | -0.05 | .43 |
| rs6311 | -0.001 | .99 | 0.01 | .86 | .01 | .96 |
| rs6323 | -0.03 | .40 | -0.01 | .91 | -.02 | .53 |

***Mother-child Model***

| Predictors | Depression symptoms | | Creativity | | Aggressive behavior | |
| --- | --- | --- | --- | --- | --- | --- |
|  | β | *p* | β | *p* | β | *p* |
| rs4570625 | -0.01 | .81 | 0.09 | .14 | -0.05 | .31 |
| rs4290270 | -0.04 | .45 | 0.02 | .36 | -0.03 | .53 |
| rs1042173 | -0.01 | .88 | 0.01 | .96 | **-0.13*** | **.02** |
| rs1364043 | -0.09 | .06 | **0.12*** | **.02** | **-0.13**** | **.01** |
| rs6295 | 0.08 | .20 | -0.11 | .06 | 0.11 | 06 |
| rs7997012 | **-0.14*** | **.04** | 0.03 | .71 | 0.05 | .44 |
| rs6311 | -0.03 | .60 | 0.05 | .34 | -0.001 | .99 |
| rs6323 | -0.02 | .56 | -0.02 | .57 | .0002 | .99 |

**Appendix 7**

**Analysis of the Main Effects of Serotonergic MGPS on Adolescent Social Adjustment**

| Predictors | Depression symptoms | | Creativity | | Aggressive behavior | |
| --- | --- | --- | --- | --- | --- | --- |
|  | β | *p* | β | *p* | β | *p* |
| rs4570625 | 0.001 | .98 | 0.07 | .08 | -0.07 | .07 |
| rs4290270 | -0.002 | .95 | 0.01 | .84 | -0.06 | .09 |
| rs1042173 | 0.03 | .48 | 0.02 | .58 | **0.09*** | **.02** |
| rs1364043 | **-0.08*** | **.04** | **-0.09*** | **.02** | 0.05 | .19 |
| rs6295 | 0.07 | .08 | 0.06 | .14 | -0.003 | .94 |
| rs7997012 | -0.04 | .31 | -0.02 | .54 | -0.04 | .27 |
| rs6311 | -0.06 | .10 | 0.03 | .51 | -0.01 | .83 |
| rs6323 | -0.04 | .26 | 0.06 | .14 | 0.01 | .70 |

**Appendix 8**

**The Effects of Independent HPA Axis System (n-1) MGPSs and Environmental Interactions on Adolescent Social Adaptation**

***Father-child Model***

| Predictors | Depression symptoms | | Creativity | | Aggressive behavior | |
| --- | --- | --- | --- | --- | --- | --- |
|  | β | *p* | β | *P* | β | p |
| -rs4570625 | **-** | **-** | **0.09*** | **.02** | **-0.06*** | **.05** |
| -rs4290270 | **-** | **-** | **0.12**** | **.001** | **-0.09**** | **.01** |
| -rs1042173 | **-** | **-** | **0.11**** | **.004** | **-0.09*** | **.02** |
| -rs1364043 | **-** | **-** | **0.13***** | **< .001** | -0.06 | .11 |
| -rs6295 | **-** | **-** | **0.08*** | **.04** | **-0.11**** | **.004** |
| -rs7997012 | **-** | **-** | **0.07*** | **.05** | **-0.09*** | **.02** |
| -rs6311 | **-** | **-** | **0.11**** | **.004** | **-0.10**** | **.004** |
| -rs6323 | **-** | **-** | 0.06 | .14 | **-0.09*** | **.01** |

***Mother-child Model***

| Predictors | Depression symptoms | | Creativity | | Aggressive behavior | |
| --- | --- | --- | --- | --- | --- | --- |
|  | β | *p* | β | *P* | β | p |
| -rs4570625 | **-0.09*** | **.02** | **0.13**** | **.001** | **-** | **-** |
| -rs4290270 | **-0.07*** | **.05** | **0.16***** | **< .001** | **-** | **-** |
| -rs1042173 | **-0.09*** | **.03** | **0.13***** | **< .001** | **-** | **-** |
| -rs1364043 | -0.05 | .22 | **0.15***** | **< .001** | **-** | **-** |
| -rs6295 | **-0.09*** | **.02** | **0.12**** | **.003** | **-** | **-** |
| -rs7997012 | -0.06 | .12 | **0.11*** | **.04** | **-** | **-** |
| -rs6311 | **-0.07*** | **.05** | **0.12**** | **.002** | **-** | **-** |
| -rs6323 | **-0.07*** | **0.5** | **0.10**** | **.01** | **-** | **-** |
